# The Blood Stage Antigen RBP2-P1 of Plasmodium vivax Binds Reticulocytes and Is a Target of Naturally Acquired Immunity

**DOI:** 10.1128/IAI.00616-19

**Published:** 2020-03-23

**Authors:** Anongruk Chim-Ong, Thitiporn Surit, Sittinont Chainarin, Wanlapa Roobsoong, Jetsumon Sattabongkot, Liwang Cui, Wang Nguitragool

**Affiliations:** a Department of Molecular Tropical Medicine and Genetics, Faculty of Tropical Medicine, Mahidol University, Bangkok, Thailand; b Department of Clinical Tropical Medicine, Faculty of Tropical Medicine, Mahidol University, Bangkok, Thailand; c Mahidol Vivax Research Unit, Faculty of Tropical Medicine, Mahidol University, Bangkok, Thailand; d Department of Internal Medicine, Morsani College of Medicine, University of South Florida, Tampa, Florida, USA; UC Davis School of Veterinary Medicine

**Keywords:** binding, immunity, invasion, reticulocyte binding protein 2-P1, vivax malaria

## Abstract

The interactions between Plasmodium parasites and human erythrocytes are prime targets of blood stage malaria vaccine development. The reticulocyte binding protein 2-P1 (RBP2-P1) of Plasmodium vivax, a member of the reticulocyte binding protein family, has recently been shown to be highly antigenic in several settings endemic for malaria. Yet, its functional characteristics and the relevance of its antibody response in human malaria have not been examined. In this study, the potential function of RBP2-P1 as an invasion ligand of P. vivax was evaluated. The protein was found to be expressed in schizonts, be localized at the apical end of the merozoite, and preferentially bind reticulocytes over normocytes. Human antibodies to this protein also exhibit erythrocyte binding inhibition at physiologically relevant concentrations. Furthermore, RBP2-P1 antibodies are associated with lower parasitemia and tend to be higher in asymptomatic carriers than in patients. This study provides evidence supporting a role of RBP2-P1 as an invasion ligand and its consideration as a vaccine target.

## Abstract (precis)

The interactions between Plasmodium parasites and human erythrocytes are prime targets of blood stage malaria vaccine development. The reticulocyte binding protein 2-P1 (RBP2-P1) of Plasmodium vivax, a member of the reticulocyte binding protein family, has recently been shown to be highly antigenic in several settings endemic for malaria. Yet, its functional characteristics and the relevance of its antibody response in human malaria have not been examined. In this study, the potential function of RBP2-P1 as an invasion ligand of P. vivax was evaluated.

## INTRODUCTION

Plasmodium vivax is the major cause of malaria outside Africa (1). All clinical manifestations of malaria are associated with the blood stage infection, during which erythrocytes are invaded by the parasite. Unlike Plasmodium falciparum, P. vivax specifically infects young erythrocytes (reticulocytes). This restricted host cell tropism is mediated by the interaction between parasite ligands and host cell receptors. Two key families of P. vivax invasion ligands are the Duffy binding protein (DBP) family and the reticulocyte binding protein (RBP) family. Cumulative evidence has strongly implicated a role of the members of the RBP family in reticulocyte selection (2–4), but proteins in the DBP family may also contribute to the host selection (5, 6).

The DBP family consists of two proteins, DBP and erythrocyte binding protein (EBP). These proteins are homologs of the P. falciparum erythrocyte binding antigens (EBAs). DBP binds the Duffy antigen receptor for chemokines (DARC), is nearly essential for P. vivax invasion of reticulocytes (7, 8), and is the most advanced candidate for a vaccine against blood stage P. vivax. EBP, on the other hand, was recently discovered (9). A recent functional study demonstrated that this protein preferentially binds Duffy-positive reticulocytes (5). The RBP family was originally discovered in 1980s, when Galinski and coworkers searched for reticulocyte-specific invasion ligands (10). Two members of the family (RBP1a and RBP2c) were discovered then. Other family members (RBP1b, RBP2a, RBP2b, RBP2-P1, and RBP2-P2) were identified when the whole-genome sequence became available, along with three pseudogenes (RBP2d, RBP2e, and RBP3) (9, 11). Most RBPs (RBP1a, RBP1b, RBP2a, RBP2b, RBP2c, and RBP2-P2) were recently characterized and found to have erythrocyte binding capacity and various degrees of reticulocyte selectivity (3, 4, 12–14). Human antibodies to some of these proteins are associated with reduced parasitemia (15) or protection against clinical disease (13). Notably, RBP2b has been shown to bind reticulocytes specifically through interactions with transferrin receptor 1 (TfR1), a surface protein abundant on the surface of reticulocytes but nearly absent from normocytes (4). The interaction between RBP2b and TfR1 was highly important for invasion; TfR1-deficient reticulocytes are highly refractory to P. vivax invasion.

The remaining uncharacterized P. vivax invasion ligand of the RBP family was RBP2-P1. With 622 amino acids, RBP2-P1 is a truncated member of the family and is approximately 4- to 5-fold smaller than the full-length RBPs. Interestingly, RBP2-P1 is composed primarily of the erythrocyte binding domain, very much like the reticulocyte-binding 5 (RH5) protein of P. falciparum. In this study, we examined the function of RBP2-P1 and its antibody response in malaria patients and asymptomatic carriers.

## RESULTS

**FIG 1:**
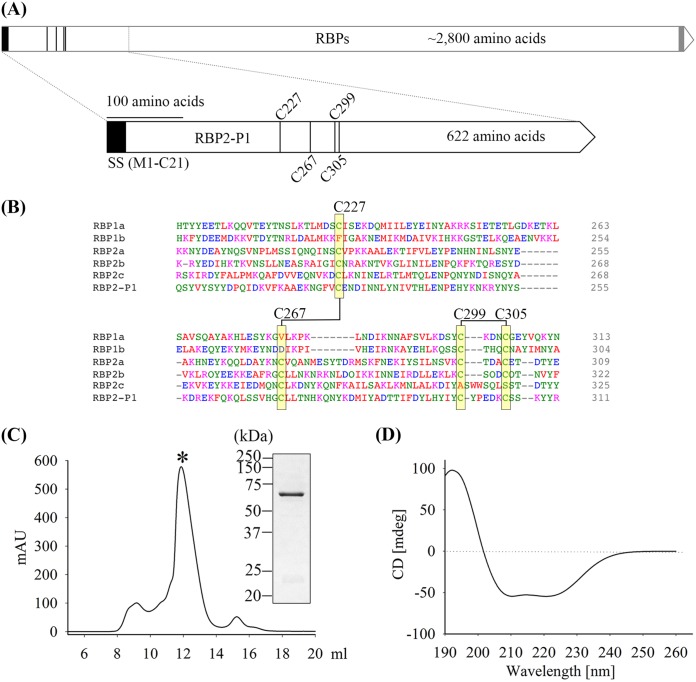
Expression of the recombinant RBP2-P1. (A) Schematic of RBP2-P1. The black block denotes the predicted signal sequence (SS; M1 to C21). The gray block indicates transmembrane domain. Long vertical lines represent the conserved cysteine residues (C227, 267, 299, and 305). (B) Alignment of a conserved domain of RBP2-P1 with other members of the P. vivax RBP family using the Sal-1 reference strain. The disulfide-bonded pairs of conserved cysteine residues are highlighted in yellow and indicated with numbers. (C) Gel filtration profile of the rRBP2-P1 and Coomassie-stained SDS-PAGE gel of the peak fraction. (D) Circular dichroism spectra of RBP2-P1.

The 622-amino-acid-long RBP2-P1 has a predicted signal peptide (residues Met1 to Cys21). Much of the sequence spans the N-terminal binding domain which is conserved among the reticulocyte binding protein family of *Plasmodium* spp. (Fig. 1A). Sequence alignment (see the supplemental material) of RBP2-P1 from 7 isolates (Sal-1, Thai, Mauritania, Brazil, India, Korea, and PVP01_0534400) reveals cysteines at positions known to form the disulfide bridges in some RBPs (12) (Fig. 1A and B).

### Expression and purification of RBP2-P1.

The recombinant RBP2-P1 (rRBP2-P1) was successfully expressed in Escherichia coli. The purified protein with the 6×His tag had an expected size of 73 kDa, consistent with the predicted molecular size of rRBP2-P1 (see Fig. S2 in the supplemental material). After removal of the 6×His tag, the final protein had a predicted size of 70 kDa and migrated as a single peak on gel filtration (Fig. 1C). Circular dichroism spectroscopy (Fig. 1D) suggests that the protein was well folded and composed mainly of alpha helices, as expected from the crystal structures of related proteins (4, 12, 17).

### RBP2-P1 is expressed in the parasite.

**FIG 2:**
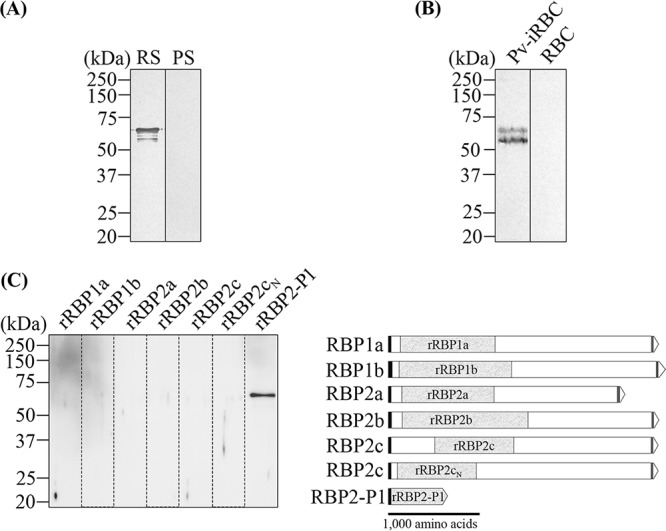
Western blots of rRBP2-P1 and P. vivax lysate. (A) rRBP2-P1 was probed by rabbit immune serum (RS) and preimmune serum (PS). (B) Rabbit immune serum recognized endogenous RBP2-P1 from P. vivax-infected erythrocytes (Pv-iRBC). Uninfected erythrocyte lysate was used as a control (RBC). (C) Rabbit immune serum recognized rRBP2-P1 but did not react with other recombinant RBPs, as follows: rRBP1a (amino acids 160 to 1170), rRBP1b (amino acids 140 to 1275), rRBP2a (amino acids 160 to 1135), rRBP2b (amino acids 161 to 1454), and rRBP2c without (2c; amino acids 501 to 1300) and with (2c_N_; amino acids 120 to 966) the binding domain.

Polyclonal antibodies against rRBP2-P1 were raised in rabbits and showed reactivity with rRBP2-P1 in immunoblots compared to no reactivity from the preimmune sera (Fig. 2A). Western blot analysis performed on P. vivax lysate using the rabbit antisera confirmed RBP2-P1 expression in the parasite. Two protein bands were detected, with the upper band having the same gel mobility as rRBP2-P1. The presence of two bands suggests proteolytic processing of the RBP2-P1 protein, as commonly observed for parasite proteins (18, 19) (Fig. 2B). The specificity of the rabbit antisera was further tested against six additional rRBPs. The results confirmed that the rabbit antisera did not cross-react with other rRBPs (Fig. 2C).

### RBP2-P1 is localized at the apical ends of merozoites.

**FIG 3:**
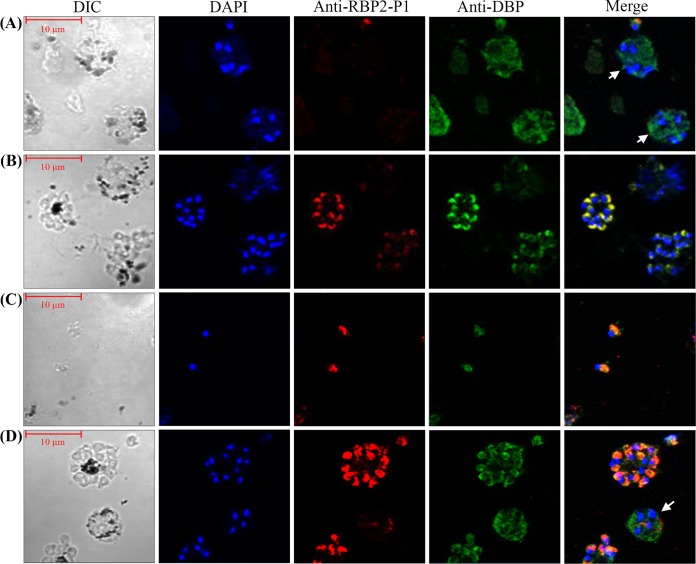
Localization of RBP2-P1 in P. vivax schizonts. (A) Early schizonts. (B) Mature schizonts. (C) Free merozoites. (D) Mixed stages. (A to D) Red, enriched schizonts probed with anti-rRBP2-P1 rabbit immune serum (A and B) or purified rabbit anti-rRBP2-P1 IgG (C and D). Green, costaining with the mouse anti-DBP antibody (DBP 3D10). Blue, DNA stained by DAPI. White arrows indicate early schizonts. DIC, differential interference contrast.

Microarray analyses of blood stage P. vivax detected peak *RBP2-P1* transcript abundance at the late schizont stage (20, 21). To determine the localization of RBP2-P1 in schizonts, an indirect immunofluorescence assay (IFA) was performed on schizonts enriched from patients’ blood samples (Fig. 3). The rabbit immune serum and anti-RBP2-P1 IgG recognized endogenous RBP2-P1 in infected red blood cells (RBCs). The fluorescent signals overlapped those of DBP, a micronemal protein, suggesting apical localization of RBP2-P1. Interestingly, the expression of RBP2-P1 was found only in late schizonts (8 nuclei or more), unlike DBP, which could already be detected in early schizonts (Fig. 3A and D).

### RBP2-P1 preferentially binds reticulocytes over normocytes.

**FIG 4:**
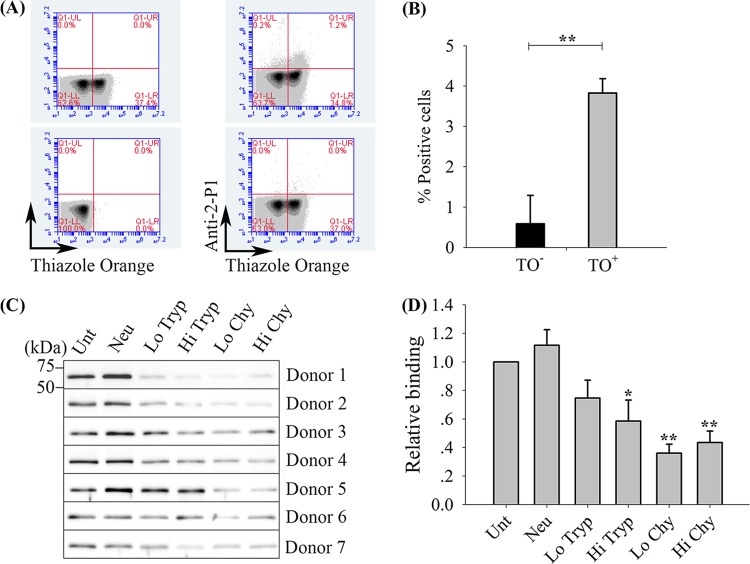
Erythrocyte binding activity of rRBP2-P1 and susceptibility of the binding to enzyme treatment. (A) Representative dot plots of rRBP2-P1 binding to erythrocytes in flow cytometry. Lower left, erythrocytes without staining. Upper left, erythrocytes stained with thiazole orange (TO) to reveal the reticulocyte subpopulation. Upper right, rRBP2-P1 binding to cells was detected by primary rabbit anti-rRBP2-P1 IgG, followed by secondary anti-rabbit Alexa 647 antibody; erythrocytes were stained with TO. Lower right, control experiment without rRBP2-P1 before incubations with primary and secondary antibodies; erythrocytes were stained with TO. (B) Bar charts showing the percentage of rRBP2-P1 binding to mature erythrocyte (TO^−^) versus reticulocyte (TO^+^) populations (*n* = 3). **, *P* < 0.001 (Student's *t* test). (C) Immunoblot of rRBP2-P1 bound to enzyme-treated erythrocytes. Seven separate experiments were performed using erythrocytes from different donors. (D) Relative binding (average of *n* = 7) of rRBP2-P1 enzyme-treated erythrocytes relative to untreated cells. Untreated, Unt; neuraminidase, Neu; low trypsin, Lo Tryp; high trypsin, Hi Tryp; low chymotrypsin, Lo Chy; high chymotrypsin, Hi Chy. *, *P* < 0.05; **, *P* < 0.001 (paired *t* test against Unt). Data are presented as the mean ± standard error of the mean (SEM).

The binding characteristics of rRBP2-P1 were determined with enriched reticulocytes using a flow cytometry-based erythrocyte binding assay (Fig. 4A). The results showed that rRBP2-P1 bound reticulocytes over normocytes with a statistically significant difference (Fig. 4B). The effect of enzyme treatment to erythrocytes on rRBP2-P1 binding was investigated by erythrocyte enzymatic treatments prior to the conventional erythrocyte binding assay (Fig. 4C). The binding activity of rRBP2-P1 was reduced when erythrocytes were pretreated with trypsin and chymotrypsin but not with neuraminidase (Fig. 4D). It is noted that while trypsin treatment generally reduced the binding of rRBP2-P1, the binding to donor 5’s cells appeared to be unaffected by trypsin. The reason for this is unclear, but it is possible that the erythrocyte receptor may carry one or more mutations that rendered the protein resistant to the enzyme.

### Erythrocyte binding of RBP2-P1 is blocked by antibodies.

**FIG 5:**
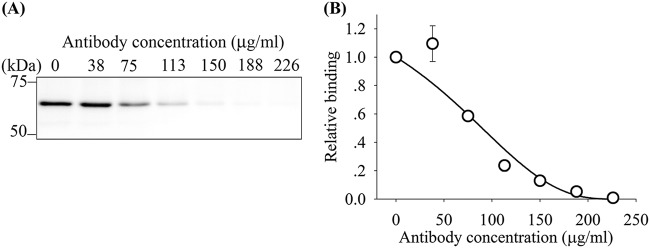
Antibodies against RBP2-P1 blocked binding of rRBP2-P1 to erythrocytes. (A) Representative immunoblot showing rRBP2-P1 binding inhibition by rabbit anti-rRBP2-P1 IgG. (B) Dose response of antibody inhibition (*n* = 3). Data are presented as the mean ± SEM.

We next investigated whether antibodies could interfere with rRBP2-P1 binding to erythrocytes. When rabbit anti-rRBP2-P1 IgG was incubated with rRBP2-P1 (5 μg/ml), binding of the rRBP2-P1 to erythrocytes was reduced in a dose-dependent manner, with a 50% inhibitory concentration (IC_50_) of 90 μg/ml (Fig. 5).

### Detection of humoral immune response against rRBP2-P1.

**FIG 6:**
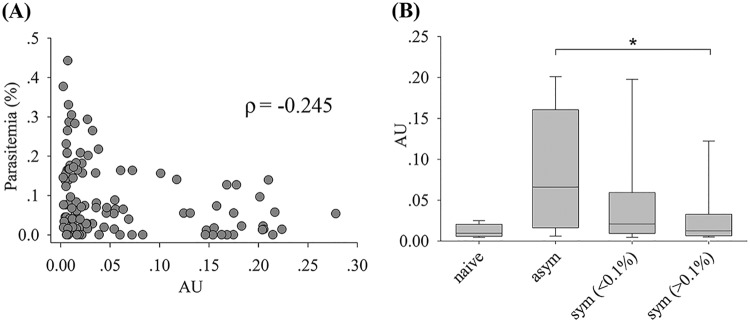
Levels of IgG to rRBP2-P1 in plasma of naive and P. vivax-infected individuals. (A) IgG levels (arbitrary units [AU]) of infected individuals as a function of parasitemia (*n* = 119). ρ, Spearman’s rank correlation. (B) IgG levels in naive individuals (*n* = 8), asymptomatic carriers (asym; *n* = 20), patients (sym) with parasitemia below 0.1% (*n* = 61), and patients with parasitemia over 0.1% (*n* = 38). *, *P* < 0.05, Mann-Whitney rank sum test.

The levels of plasma IgG against rRBP2-P1 were measured using an enzyme-linked immunosorbent assay (ELISA) with samples from 127 individuals from an area of endemicity in western Thailand, as follows: 99 P. vivax malaria patients, 20 asymptomatic carriers, and 8 naive individuals (Fig. 6). Patient samples were obtained from malaria clinics. Asymptomatic carriers were identified from a previous 2013–2014 cohort study in western Thailand (22). They were villagers who were P. vivax positive at multiple times (3 to 13 times) out of the 14 monthly visits but never developed any malaria-like symptoms. Naive individuals were people from the same study area who were not infected during the study period and reported not to have had malaria before. The general characteristics of the study participants are provided in Table 1. The levels of RBP2-P1 antibodies in patients and asymptomatic carriers were negatively correlated with parasitemia (*P = *0.007, Spearman’s rank correlation) (Fig. 6A). This relationship remains statistically significant after age has been taken into account (*P < *0.01, multivariate linear regression). Consistently, asymptomatic carriers tend to have higher antibody levels than do patients with parasitemia above 0.1% (Fig. 6B) (*P = *0.012, Mann-Whitney rank sum test).

**TABLE 1:** Demographic and infection characteristics of P. vivax-infected participants

Characteristic	Value^*a*^
Sex	
Female	37
Male	82
Age (yr)	
15–20	14
21–40	63
41–60	38
>60	4
Parasitemia	
<0.01%	21
0.01–0.1%	60
>0.1%	38
Type of infection	
Asymptomatic	20
Clinical	99
RBP2-P1 antibody (median [minimum–maximum]) (AU)	0.020 (0.0025–0.28)

a Values are numbers of participants with the associated characteristic, unless otherwise indicated.

### P. vivax patient antibodies inhibit rRBP2-P1 binding to erythrocytes.

**FIG 7:**
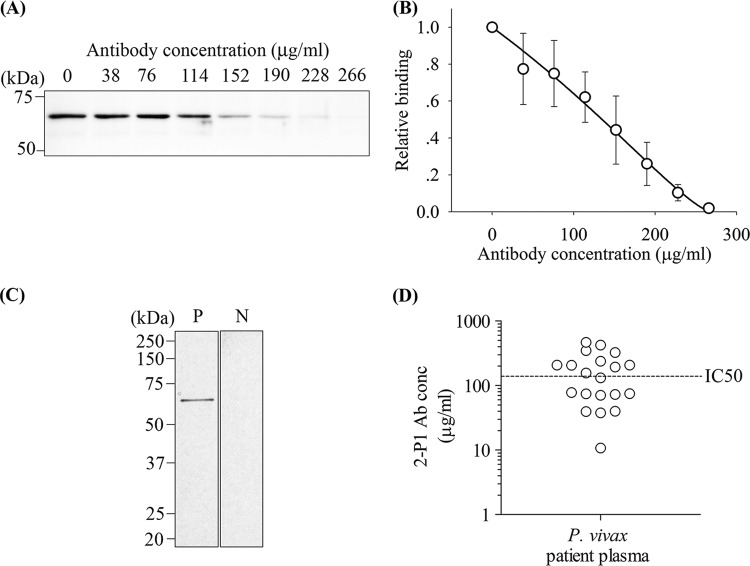
Human anti-RBP2-P1 antibodies recognized rRBP2-P1 and inhibited its erythrocyte binding activity. (A) Representative immunoblot showing rRBP2-P1 binding inhibition by purified human anti-RBP2-P1 antibodies. (B) Dose response of antibody inhibition (*n* = 3). Data are presented as the mean ± SEM. (C) Immunoblots of rRBP2-P1 with patient serum (P) or naive serum (N) as primary antibodies. (D) Concentration (conc) of anti-RBP2-P1 antibody (Ab) in patient plasma. Only plasma samples from 20 patients with highest antibody response in Fig. 6A were analyzed.

In agreement with the observation that P. vivax malaria patients had higher IgG levels against rRBP2-P1 than did naive individuals, immunoblots showed that patient serum, but not naive serum, reacted with the rRBP2-P1 protein (Fig. 7C). To test whether human antibodies could inhibit erythrocyte binding of rRBP2-P1, we affinity-purified immunoglobulins recognizing rRBP2-P1 from pooled plasma from 10 vivax malaria patients and used them in the binding inhibition assay. Similar to the rabbit anti-rRBP2-P1 IgG, the purified human antibodies could inhibit the binding with an IC_50_ of 135 μg/ml (Fig. 7A and B), a level that can be attained in some human patient plasma (Fig. 7D).

## DISCUSSION

RBP2-P1 was discovered when the first P. vivax genome was sequenced (11). Interestingly, RBP2-P1 was first annotated as a pseudogene, perhaps reflecting its unusual structural architecture containing only the N-terminal binding domain. Among the RBPs, only RBP2-P1 and RBP2-P2 have this truncated structure, which is shared by PfRH5, an essential invasion ligand of P. falciparum which interacts with human basigin on the surface of erythrocytes. Whereas RBP-2P2 is absent in some isolates, RBP2-P1 is found in all P. vivax isolates (11, 15, 23), as well as in Plasmodium cynomolgi (24). These observations together suggest the importance of RBP2-P1. Until now, RBP2-P1 has remained the only P. vivax RBP that has not been functionally examined.

We successfully expressed full-length rRBP2-P1 and characterized its binding to erythrocytes. Like several RBPs (3, 13, 14), the protein preferentially binds reticulocytes over normocytes. Binding to erythrocytes was sensitive to trypsin and chymotrypsin treatments but insensitive to neuraminidase treatment. This sensitivity profile differs from that of PfRH5, suggesting that RBP2-P1 may not bind basigin. In contrast, the enzyme sensitivity profile of the rRBP2-P1-erythrocyte interaction was similar to that of PfRH4, which binds the human complement receptor 1 (CR1) (25), and RBP2b, which binds human transferrin receptor 1 (TfR1) (4). The erythrocyte receptor of RBP2-P1 is currently not known. It is also unclear whether this protein functions as one of the alternate invasion pathways among the RBPs. However, the fact that it has a distinct structure, being the smallest and composed only of the N-terminal domain, suggests that it has a unique function.

Natural infection of P. vivax has recently been shown to induce strong antibody response to RBP2-P1 (26). In the current study, the levels of human antibody to RBP2-P1 were measured in both P. vivax patients and carriers. Higher responses were associated with lower parasite densities. Consistently, the levels of anti-rRBP2-P1 antibodies are generally higher in asymptomatic carriers than in patients, a trend shared by several blood stage vaccine targets, including P. falciparum EBAs (PfEBAs), P. falciparum merozoite surface proteins (PfMSPs), and PfRHs (27, 28). Although the association may reflect past exposure, the fact that human antibodies interfere with RBP2-P1 erythrocyte binding suggests that these antibodies could contribute to protection by inhibiting erythrocyte invasion. An invasion-blocking experiment will be required to test this possibility. It is also possible that RBP2-P1 antibodies provide clinical protection through a different mechanism, such as complement fixation (29).

In summary, this study provides data supporting the presence and role of RBP2-P1 as a reticulocyte selective invasion ligand of P. vivax. Natural antibodies to this protein can inhibit erythrocyte binding and are associated with protection. Thus, this protein appears to be an attractive target for development of a blood stage vaccine against P. vivax.

## MATERIALS AND METHODS

### Ethics statement.

The use of human specimens in study was approved by the ethics committee of the Faculty of Tropical Medicine at Mahidol University.

### Cloning, expression, and purification of recombinant RBP2-P1.

The sequence of RBP2-P1 was retrieved from the GenBank (accession no. KU158839). The gene without the predicted signal peptide was amplified from genomic DNA of the Thai P. vivax isolate VTTY57 using primers 5′-AATAAAggatccCGAAGCAAACCTAGCAGACTAAA-3′ and 5′-TATATTctcgagTTAGAATGGCTTTAATATTTTATTAACATC-3′ (restriction sites for cloning into expression plasmid are shown in lowercase). The PCR product encoding amino acids 26 to 622 was digested with BamHI and XhoI (New England BioLabs) and inserted downstream of the T7 promoter in the E. coli expression vector pPROEX HTb (Life Technologies). The encoded protein has an N-terminal 6-histidine (6×His) tag and a tobacco etch virus (TEV) protease cleavage site to remove the 6×His tag. Sequencing of the plasmid confirmed the correct sequence and reading frame of the RBP2-P1 gene.

The recombinant RBP2-P1 (rRBP2-P1) was expressed in 2 liters of E. coli SHuffle T7 cells (New England BioLabs) by induction with 0.5 mM isopropyl-β-d-1-thiogalactopyranoside (IPTG). For protein purification, a cell pellet was suspended in a lysis buffer (300 mM NaCl, 50 mM Tris-HCl, 5 mM imidazole [pH 7.7], 10% [vol/vol] glycerol) with 1.0 mg/ml lysozyme (Sigma). The protease inhibitor phenylmethylsulfonyl fluoride (PMSF) was added to a final concentration of 1 mM immediately before sonication. The resulting cell lysate was centrifuged at 20,000 × *g* for 30 min at 4°C. The supernatant was filtered through a 0.45-μm polyvinylidene difluoride (PVDF) filter (Merck) before protein capturing by cobalt (Co^2+^) resins (Talon) prewashed with lysis buffer. The unbound material was washed away with 20 column volumes of the washing buffer (300 mM NaCl, 20 mM Tris-HCl, 5 mM imidazole [pH 7.7], 10% [vol/vol] glycerol), followed by 2.5 column volumes of the washing buffer supplemented with additional 5 mM imidazole. Bound protein was eluted with 10 column volumes of the elution buffer (300 mM NaCl, 20 mM Tris-HCl, 350 mM imidazole [pH 7.7], 10% [vol/vol] glycerol). The eluted fraction was concentrated and injected into a Superdex 200 Increase 10/300 GL (GE Healthcare) fast-performance liquid chromatography (FPLC) column preequilibrated with phosphate-buffered saline (PBS; pH 7.4). The single-peak fraction containing pure protein was collected and the protein concentration determined by measuring the absorbance at 280 nm (*A*_280_), assuming 1 absorbance unit to be equal to 1 mg/ml. Circular dichroism spectroscopy was used to examine the secondary structure of the protein.

When removal of the 6×His tag was needed, 2 mg TEV protease was added per 10 mg concentrated recombinant protein after elution from the Co^2+^ column. The mixture was dialyzed twice in a 100-fold volume of dialysis buffer (300 mM NaCl, 20 mM Tris-HCl [pH 7.7], 10% [vol/vol] glycerol). NaCl was added to bring the final NaCl concentration to 500 mM, and the protein was captured by passing through 4-ml Co^2+^ resins prewashed and equilibrated with the dialysis buffer. The final recombinant protein without the 6×His tag was eluted with 20 column volumes of the washing buffer, concentrated, and subjected to FPLC as described above. The protein was stored at –80°C.

### SDS-PAGE and Western blot analysis.

rRBP2-P1 (without a 6×His tag) (1 μg) was separated by a 10% SDS-PAGE gel and stained with Coomassie blue. For Western blot analysis, the protein (with a 6×His tag) was loaded in each lane, electrophoresed, and transferred to PVDF membranes (Millipore). The membrane was blocked with blocking buffer (5% skim milk in PBS containing 0.1% Tween 20 [PBST]) for 4 h at room temperature (RT) and incubated with anti-His tag monoclonal antibody, clone HIS.H8, at a 1:2,000 dilution (Millipore), or the TEV cleavage site monoclonal antibody at a 1:1,000 dilution (Thermo Fisher Scientific) in the blocking buffer overnight at 4°C. After membrane washes with PBST, the membrane was incubated with goat anti-mouse horseradish peroxidase (HRP; 1:5,000 dilution; Millipore) in the blocking buffer for 1 h at RT. The membrane was washed, and the HRP activity was detected using the 3,3′,5,5′-tetramethylbenzidine reagent (Millipore). To determine the reactivity of patient serum, rRBP2-P1 (without a 6×His tag) was probed with patient serum (1:250 dilution) and incubated with the goat anti-human HRP (1:5,000 dilution; Millipore). The reactivity of rabbit immune serum to the parasite protein was determined with parasite lysate. A total of ∼3 × 10^9^ cells of P. vivax-infected RBCs were scrapped from thin-film blood smears and added to 4× reducing sample loading buffer. The membrane containing parasite protein was probed with rabbit immune serum (1:1,000 dilution) and then incubated with the goat anti-rabbit HRP (1:5,000 dilution; Millipore).

### Antibody production.

Antibodies against rRBP2-P1 were produced by Thermo Fisher Scientific (USA). Briefly, two rabbits were immunized intramuscularly with 0.50 mg of rRBP2-P1 emulsified with Freund’s complete adjuvant on day 0, followed by three boosts of the antigen emulsified with incomplete Freund’s adjuvant on days 14, 42, and 56. The serum was collected on day 70. Serum was also used for rabbit IgG purification by protein G Sepharose (GE Healthcare), according to the standard protocol. The specificity of serum for rRBP2-P1 was confirmed by Western blotting against recombinant P. vivax RBPs (rRBPs) (13). For this, 1 ng of each rRBP was separated on SDS-PAGE gels and transferred to the membrane. The membrane containing proteins was probed with rabbit anti-rRBP2-P1 IgG (1 μg/ml), followed by goat anti-rabbit HRP (0.2 μg/ml; Millipore). Reactions were detected with chemiluminescence. Coomassie-stained SDS-PAGE of the same rRBP samples (1 μg/lane) was used to demonstrate that each sample contained a protein (see Fig. S1 in the supplemental material).

### Indirect immunofluorescence assay.

Blood smears containing enriched late-stage P. vivax from a Thai patient were fixed with ice-cold acetone, blocked with PBS containing 3% bovine serum albumin (BSA) at 37°C for 30 min, and washed three times with PBS. Rabbit immune serum at a 1:200 dilution or purified rabbit anti-RBP2-P1 IgG at 1.3 μg/ml was used as the primary antibody and incubated at 37°C for 1 h. After three washes with PBS, the slides were stained with a combination of goat anti-mouse Alexa Fluor 568 at a 1:500 dilution, mouse anti-DBP (DBP 3D10) Alexa Fluor 488 at a 1:100 dilution, and 4′,6′-diamidino-2-phenylindole (DAPI; Invitrogen) at a 1:1,000 dilution and incubated at 37°C for 30 min. Finally, the slides were mounted with ProLong Gold antifade reagent (Invitrogen) and visualized under a confocal microscope (Zeiss LSM700) equipped with a 100× oil objective. Images were captured using the ZEN software (Zeiss).

### Affinity purification of RBP2-P1-specific human antibodies.

For affinity purification of human antibodies, rRBP2-P1 (10 mg) was coupled to 1 ml packed cyanogen bromide (CNBr)-activated Sepharose (GE Healthcare), according to the manufacturer’s instruction. Ten milliliters of serum samples pooled from 10 P. vivax malaria patients was used for antibody purification. The pooled sera were centrifuged to remove insoluble particles before incubating with the rRBP2-P1-coupled resins for 1 h at RT. The unbound materials were washed away with 10 column volumes of PBS. Bound antibodies were eluted with 100 mM glycine (pH 3.0) into 1.0 M Tris-HCl (pH 8.0) neutralizing solution. The buffer was then exchanged to PBS. Antibody concentration was estimated using the Bradford assay.

### Reticulocyte enrichment.

Reticulocytes were enriched from whole blood from the Thai Red Cross Society. Blood was passed through a leukocyte reduction filter (Haemonetics Corporation) and centrifuged at 1,000 × *g* for 10 min to discard the plasma. The packed cells were mixed with 2 volumes of incomplete RPMI 1640 medium and centrifuged at 1,000 × *g* for 10 min. The packed cells were diluted to 20% hematocrit. Then, 4 ml of the diluted blood was gently layered on top of 4 ml of 15% OptiPrep-KCl (Axis-Shield) and centrifuged at 3,000 × *g* for 30 min without braking. Reticulocytes were collected at the interface, washed 3 times with incomplete RPMI 1640 medium, and kept at 4°C.

### Flow cytometry erythrocyte binding assay.

A flow cytometry-based quantitative erythrocyte binding assay was performed as follows. First, an enriched reticulocyte fraction was washed three times with PBS and incubated in the blocking solution (1% BSA in PBS containing 0.6 mM CaCl_2_) at room temperature for 1.5 h. After centrifugation and removal of blocking solution, 100 μl of rRBP2-P1 solution (5 μg in PBS plus 0.6 mM CaCl_2_) or control solution (PBS plus 0.6 mM CaCl_2_) was added to 1 × 10^7^ cells and incubated at room temperature. After 1.5 h of incubation, cells were centrifuged, and the supernatant was removed. Rabbit anti-RBP2-P1 IgG (10 μg in 200 μl blocking solution) was added to the cells. After 1 h, the suspension was centrifuged and the supernatant removed. Two hundred microliters of Alexa Fluor 647 chicken anti-rabbit secondary antibody (Invitrogen; 1:200 dilution in blocking solution) was added and incubated for 1 h at room temperature in the dark. The suspension was then centrifuged and the supernatant removed. Finally, 200 μl of thiazole orange (TO) solution (BD Retic-Count reagent; BD Biosciences) supplemented with 0.6 mM CaCl_2_ was added. The cell suspension was incubated at room temperature for 30 min in the dark, and 500,000 erythrocytes were read and analyzed on an Accuri C6 flow cytometer (BD Biosciences).

### Conventional erythrocyte binding assay.

The conventional erythrocyte binding assay was performed using a standard procedure (16). Briefly, 100 μl of erythrocytes was washed three times with PBS, and the resulting packed cells were mixed with 100 μl PBS containing rRBP2-P1 (0.5 μg for protein without 6×His tag, or 10 μg for protein with 6×His tag) and incubated for 1.5 h at RT. The cell suspension was then centrifuged through 500 μl dibutyl phthalate (Sigma) at 12,000 × *g* for 30 s. The supernatant and oil were removed, and the packed cells were centrifuged once again through dibutyl phthalate. Bound protein was eluted from packed erythrocytes with 50 μl of 1.5 M NaCl. The presence of recombinant protein in the eluate was determined by Western blotting using rabbit anti-RBP2-P1 IgG or anti-His tag antibody as the primary antibody, followed by appropriate horseradish peroxidase (HRP)-conjugated secondary antibodies for chemiluminescent detection using Immobilon Western substrates (Millipore) and detected with the GeneSys image capture software (G:BOX Chemi XRQ Syngene). The densitometry analysis was performed using the ImageQuant TL software (GE Healthcare).

For enzymatic treatment, erythrocytes at 10% hematocrit in PBS were treated with 66 mU/ml neuraminidase (Sigma) or with trypsin/chymotrypsin (Sigma) at 0.2 and 2 mg/ml as low- and high-concentration enzymatic treatments, respectively, at 37°C for 1 h. The treated cells were washed once with 10 volumes of PBS and incubated with 10 volumes of 2 mM phenylmethylsulfonyl fluoride (PMSF) in PBS at RT for 15 min. After three washes with 10 volumes of PBS, the treated cells were used immediately in erythrocyte binding assays.

To determine whether RBP2-P1 antibodies could block *in vitro* binding of the RBP2-P1 protein to erythrocytes, rRBP2-P1 (without 6×His tag, 0.5 μg) was mixed with 3.8, 7.5, 11.3, 15.0, 18.8, and 22.6 μg of rabbit anti-RBP2-P1 IgG or 3.8, 7.6, 11.4, 15.2, 19.0, 22.8, and 26.6 μg human anti-RBP2-P1 antibodies in a final volume of 100 μl. The mixture was incubated for 1 h at RT and transferred into 100 μl packed erythrocytes. The binding of rRBP2-P1 to erythrocytes was determined as described above. The presence of bound protein was determined by Western blotting using HRP-conjugated rabbit anti-RBP2-P1 IgG for direct chemiluminescent detection and quantified by densitometry. The dose-response curves (Fig. 5 and 7) were fitted with Hill’s equation for the binding of rRBP2-P1 to erythrocytes, assuming that the rRBP2-P1 free concentration is reduced linearly by the increasing concentration of antibodies.

### Enzyme-linked immunosorbent assay.

An ELISA was used to detect RBP2-P1-specific antibodies in human plasma samples. Briefly, 96-well flat-bottom plates (Nunc) were coated with 1 μg of rRBP2-P1 in PBS overnight at 4°C. In each plate, a control well with no protein was included for each individual plasma sample. Coated plates were washed 3 times with 200 μl PBST and blocked with 10% skim milk in PBST for 1 h at RT. Plasma samples were diluted 1:1,000 in PBST plus 1% skim milk, and 100 μl was used per well. The plates were incubated at RT for 1 h and washed 3 times with 200 μl PBST. For total IgG detection, 100 μl of HRP-conjugated goat anti-human IgG at 1:2,000 dilution in PBST plus 1% skim milk was added and incubated for 1 h at RT. Plates were then washed 3 times with PBST and 3 times with PBS. HRP activity was detected with 2,2′-azino-bis(3-ethylbenzthiazoline-6-sulfonic acid) (Millipore) by tracking the optical density (OD) at 405 nm every minute for 45 min. An antibody unit (AU) was the slope of the OD between 5 and 10 min. To determine the absolute concentration (in micrograms per milliliter) of anti-RBP2-P1 antibody in selected plasma samples, a standard curve was prepared by 2-fold serial dilutions of affinity purified human anti-RBP2-P1 antibodies. The concentration range of the antibodies in the standard curve was 20 to 600 μg/ml.

### Statistical analysis.

Statistical analyses were performed using SigmaPlot (Systat Software, Inc.) and PASW Statistics 18 (SPSS). The results of binding assays represent the averages from at least three independent experiments, and the error bars represent the standard errors of the mean. A comparison of means was made by Student’s and paired *t* tests. The antibody levels of malaria patients and parasite carriers were compared using the Mann-Whitney rank sum test. Sex, age, and the RBP2-P1 antibody level were examined as potential factors underlying parasitemia. Only the antibody level was found to be a significant determinant of parasitemia in univariate analysis (*P = *0.004 in linear correlation and 0.007 in rank correlation). The relationship between the antibody level and parasitemia remained significant (*P* = 0.003) when all three variables (sex, age, and antibody level) were included in a multivariate linear regression model. The raw data used in these analyses are provided in Table S1.

## Supplementary Material

Supplemental file 1
