# Supplementary material for: The Blood Stage Antigen RBP2-P1 of Plasmodium vivax Binds Reticulocytes and Is a Target of Naturally Acquired Immunity
Source: Infect Immun. 2020 Mar 23;88(4):e00616-19. doi: 10.1128/IAI.00616-19 (PMC7093139; doi:10.1128/IAI.00616-19)
Supplement: Supplemental file 1 [file IAI.00616-19-s0001.pdf]

Supplemental Material for "The blood stage antigen RBP2-P1 of *Plasmodium vivax* binds reticulocytes and is a target of naturally acquired immunity" (Chim-Ong, et al.)

This supplemental file is organized into three sections, including

1. Supplemental Figures: Fig. S1 and Fig. S2 (page 2)

2. Sequence alignment of RBP2-P1 from 7 *P. vivax* isolates (page 3-5)

3. Supplemental Table S1 (page 6 – 9)

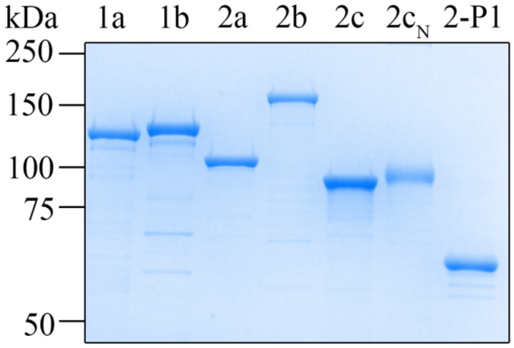

FIG S1. Recombinant RBPs (1 μg/lane) were resolved by 7.5% SDS-PAGE and stained with Coomassie Brilliant Blue. The predicted protein sizes are 118 kDa (rRBP1a, 1a); 133 kDa (rRBP1b, 1b); 114 kDa (rRBP2a, 2a); 153 kDa (rRBP2b, 2b); 94 kDa (rRBP2c without binding domain, 2c); 94 kDa (rRBP2c with binding domain, $2c_N$); and 70 kDa (rRBP2-P1, 2-P1). Also indicated are the locations of protein molecular mass markers (kDa).

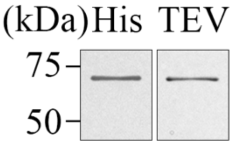

FIG S2. Immunoblots of rRBP2-P1. Purified soluble recombinant rRBP2-P1 was resolved by 12% SDS-PAGE and probed with anti-6-His antibodies (His) or the TEV cleavage site antibodies (TEV) present on the N-terminus of the protein.

Sequence alignment of RBP2-P1 from 7 *P. vivax* isolates

Predicted coding sequences of RBP2-P1 were retrieved http://www.plasmodb.org (Sal-1), http://www.broadinstitute.org/annotation/genome/plasmodium_vivax/MultiHome.html (Mauritania, Brazil, India, and Korea) with Gene identifier: PVX_090330, GenBank (Thai) with accession number: KU158839 and www.pnas.org/cgi/doi/10.1073/pnas.1810053115 (PVP01_0534400). Alignment was generated by Clustal Omega (https://www.ebi.ac.uk/Tools/msa/clustalo/).

An asterisk (*) represents identical residues. A colon (:) indicates substitutions. A dot (.) denotes semi-conserved substitutions.

```
Sal-1            MEKNILWVIFYNFLFILLASCKDTHRSKPSRLKHDHNLLPNYANLMRDDQNGQNSENRGD 60
PVP01_0534400    MEKNILWVIFYNFLFILLASCKDTHRSKPSRLKHDHNLLPNYANLMRDDQNGQNSEIRGD 60
Korea            ---------------------------------------------MKDDQNGQNSENRGD 15
Brazil           MEKNILWVIFYNFLFILLASCKDTHRSKPSRLKHDHNLLPNYANLMRDDQNGQNSENRGD 60
Mauritania       MEKNVLWVIFYNFLFILLASCKDTHRSKPSRLKHDHNLLPNYVNLMKDDQNGQNSENRGD 60
India            MEKNILWVIFYNFLFILLASCKDTHRSKPSRLKHDHNLLPNYVNLMKDDQNGQNSENRGD 60
Thai             MEKNILWVIFYNFLFILLASCKDTHRSKPSRLKHDHNLLPNYVNLMKDDQNGQNSENRGD 60
                                                              *:********* ***

Sal-1            NINNHNKNHNDQNNHNGNNDNSINSEYLKTSHLQNSSAMVHLNDHKITTKPARYSYIQRS 120
PVP01_0534400    NINNHNKNHNDQNNHNGNNDNSINSEYLKTSHLQNSSAMVHLNDHKITTKPARYSYIQRS 120
Korea            NINNHNKNHNDQNNHNGNNDNSINSEYLKTSHLQNSSAMVHLNDHKITTKPARYSYIQRS 75
Brazil           NINNHNKNRNDQNNHNGNNDNSINSEYLKTSHLQNSSAMVHLNDHKITTKPARYSYIQRS 120
Mauritania       NINKPNKNHNDQNNHNGNNDNSINSEYLKTSHLQNSSAMVHLNDHKITTKPARYSYIQRS 120
India            NINNHNKNHNDQNNHNGNNDNSINSEYLKTSHLQNSSAMVHLNDHKITTKPARYSYIQRS 120
Thai             NINNHNKNHNDQNNHNGNNDNSINSEYLKTSHLQNSSAMVHLNDHKITTKPARYSYIQRS 120
                 ***: ***:***************************************************

Sal-1            KIYAFNPNNKKIENINNELHSVPNSFVQYYVYTNLNVIVQKMRYISIIDEYNNIISLHLP 180
PVP01_0534400    KIYAFNPNNKKIENINNELHSVPNSFVQYYVYTNLNVIVQKMRYISIIDEYNNIISLHLP 180
Korea            KIYALNPNNKKIENINNELHSVPNSFVQYYVYTNLNATVYKMRYISIFDEYNNIVSLHLP 135
Brazil           KIYALNPNNKNIEKINNELHTVPNSFVQYYVYTNLNDTVYKMRYISIFDEYNNIVSLHLP 180
Mauritania       KIYALNPNNKKIENINNELHSVPNSFVQYYVYTNLNATVYKMRYISIFDEYNNIVSLHLP 180
India            KIYALNPNNKKIENINNELHSVPNSFVQYYVYTNLNATVYKMRYISIFDEYNNIVSLHLP 180
Thai             KIYALNPNNKKIENINNELHSVPNSFVQYYVYTNLNATVYKMRYISIFDEYNNIVSLHLP 180
                 ****:*****:**:******:***************  * *******:******:*****

Sal-1            YHARVYYLMELKNYSVAHKELQSYVYSYYDPQIDKVFKAAEKNGFVCENDINNLYNIVTH 240
PVP01_0534400    YHARVYYLMELKNYSVAHKELQSYVYSYYDPQIDKVFKAAEKNGFVCENDINNLYNIVTH 240
Korea            YHARVNYLMEIKNYSVAYRELQSDVYSYYDPQIDKVFKVAEKNAIVCEKYINNLYNTVTR 195
Brazil           YHARVNYLMEIKNYSVAYRELQSDVYSYYDPQIDKVFKVAEKNAIVCEKYINNLYNTVTR 240
Mauritania       YHARVNYLMEIKNYSVAYRELQSDVYSYYDPQIDKVFKVAEKNAIVCESNIKNLYNTVTR 240
India            YHARVNYLMEIKNYSVAYRELQSDVYSYYDPQIDKVFKVAEKNAIVCEKYINNLYNTVTR 240
Thai             YHARVNYLMEIKNYSVAYRELQSDVYSYYDPQIDKVFKVAEKNAIVCEKYINNLYNTVTR 240
                 ***** ****:******::**** **************.****.:***. *:**** **:
```

```
Sal-1            LENPEHYKNKRYNYSKDREKFQKQLSSVHGCLLTNHKQNYKDMIYADTTIFDYLHYIYCY 300
PVP01_0534400    LENPEHYKNNRYNYSKDREKFQKQLSSVHGCLLTNHKQNYKDMIYADTTIFDYLHYIYCY 300
Korea            LENPEQFKNNRDNYSKEIKEYQKQLTSLHDCLFENHRQNYKDIIYADTTIFDSLQNIYCY 255
Brazil           LENPEQFKNNRDNYSKEIKEYQKQLTSLHDCLFKNHRQNYKDIIYADTTIFDSLQNIYCY 300
Mauritania       LENPEQFKNNRDNYSKEIKEYQKQLTSLHDCLFKNHRQNYKDIIYADTTIFDSLQNIYCY 300
India            LENPEQFKNNRDNYSKEIKEYQKQLTSLHDCLFENHRQNYKDIIYADTTIFDSLQNMYCY 300
Thai             LENPEQFKNNRDNYSKEIKEYQKQLTSLHDCLFENHRPNYKDIIYADTTIFDSLQNIYCY 300
                 *****::**:* ****: :::****:*:*.**: **: ****:********* *: :***

Sal-1            PEDKCSSKYYRDMLNISMGKINEYEQKKKNEAINKIIDVHETGAYVMRKIKEELNPSLNS 360
PVP01_0534400    PEDKCSSKYYRDMLNISMGKINEYEQKKKNEAINKIIDVHETGAYVMRKIKEELNPSLNS 360
Korea            SEDKCLSKLYRDMLGLSMKKINEYEQKKKNEDINKIINIHDTAVDVMRKIKRELKPSFDS 315
Brazil           SEDKCLSKLYRDMLGLSMKKINEYEQKKKNEDINKIINIHDTAVDVMRKIKRELKPSFDS 360
Mauritania       SEDKCLSQLYRDMLGLSMKKINEYEQKKKNEDINKIINIHDTAVDVMRKIKRELKPSFDS 360
India            SEDKCLSKLYRDMLGLSMKKINEYEQKKKNEDINKIINIHDTAVDVMRKIKRELKPSFDS 360
Thai             SEDKCLSKLYRDMLGLSMKKINEYEQKKKNEDINKIINIHDTAVDVMRKIKRELNPSLNS 360
                  **** *: *****.:** ************ *****::*:*.. ******.**:**::*

Sal-1            DVADFVIDEIKYIIERLNAHSEKIKCASDFIKHIYKEKVQNEISKNELESNYIVLSIHHG 420
PVP01_0534400    DVADFVIDEIKYIIERLNAHSEKIKCASDFIKHIYKEKVQNEISKNELESNYIVLSIHHG 420
Korea            DTADFVIGEIKYIIERLKAHSEKIKSASDLVKYINQETVPNEISKNEIKSNYIVLAIHTG 375
Brazil           DTADFVIGEIKYIIERLKAHSEKIKSASDLVKYINQETVPNEISKNEIKSNYIVLAIHTG 420
Mauritania       DTADFVIGEIKYIIERLKAHSEKIKSASDLVKYINQETVPNEISKNEIKSNYIVLAIHTG 420
India            DTADFVIGEIKYIIERLKAHSEKIKSASDLVKYINQETVPNEISKNEIKSNYIVLAIHTG 420
Thai             DVADFVIGEIKYIIERLKAHSEKIKSASDLVKYINQETVPNEISKNEIKSNYIVLAIHTG 420
                 *.*****.*********:*******.***::*:* :*.* *******::******:** *

Sal-1            SFRFSTEHVIMLEEIFKSKEQILHNICSKFLNELKNKITALINSEYSSSKCTPIVSTCEE 480
PVP01_0534400    SFRFSTEHVIMLEEIFKSKEQILHNICSKFLNELKNKITALINSEYSSSKCTPIVSTCEE 480
Korea            SFLFSTEHVKMLEEIFKSKEQILYKICSKFLNDLKNRITTLINSEYSSSNCTPIVSTCEE 435
Brazil           SFLFSTEHVKMLEEIFKSKEQILYKICSKFLNDLKNRITTLINSEYSSSNCTPIVSTCEE 480
Mauritania       SFLFSTEHVKMLEEIFKSKEQILYKICSKFLNDLKNRITTLINSEYSSSNCTPIVSTCEE 480
India            SFLFSTEHVKMLEEIFKSKEQILYKICSKFLNDLKNRITTLINSEYSSSNCTPIVSTCEE 480
Thai             SFLFSTEHVKMLEEIFKSKEQILYKICSKFLNDLKNRITTLINSEYSSSNCTPIVSTCEE 480
                 ** ****** *************::*******:***:**:*********:**********

Sal-1            AKTSLESLRTSSTEKLGKHDLNSKPEIASVKQSYDGKMIKLAEAIKRAEEIINSVKDIVQ 540
PVP01_0534400    AKTSLESLRTSSTEKLGKHDLNSKPEIASVKQSYDGKMIKLAEAIKRAEEIINSVKDIVQ 540
Korea            AKKSLESLRTSSTGKLGNRDLNSKSEIASVKQSYDGKMLKLEEAIKRAEEIINSVNEIVQ 495
Brazil           AKKSLESLRTSSTGKLGNRDLNSKSEIASVKQSYDGKMIKLAEAIKRAEEIINSVKDIVQ 540
Mauritania       AKKSLESLRTSSTGKLGNRDLNSKSEIASVKQSYDGKMIKLAEAIKRAEEIINSVKDIVQ 540
India            AKKSLESLRTSSTGKLGNRDLNSKSEIASVKQSYDGKMLKLEEAIKRAEEIINSVNEIVQ 540
Thai             AKKSLESLRTSSTGKLGKHDLNSKPEIASVKQSYDGKMIKLAEAIKRAEEIINSVKDIVQ 540
                 **.********** ***::***** *************:** *************::***

Sal-1            FNTTETDTMKKETDRIISLNINPLSKDKMLLEVIDSIKKQKEKISENSNKIKESSDAADA 600
PVP01_0534400    FNTTETDTMKKETDRIISLNINPLSKDKMLLEVIDSIKKQKEKISENSNKIKESSDAADA 600
Korea            FNTTETEAKKKETDS-IPQNINALEKDKKLLEVIDAIKKQKQKISENSNKIKEFSGAADT 554
Brazil           FNTTETDTMKKETDRIISLNINALEKDKKLLEVIDAIKKQKQKISENSNKIKEFSGAADT 600
Mauritania       FNTTETEAKKKETDS-IPQNINALEKDKKLLEVIDAIKKQKQKISENSNKIKEFSGAADT 599
India            FNTTETEAKKKETDS-IPQNINALEKDKKLLEVIDAIKKQKQKISENSNKIKEFSGAADT 599
Thai             FNTTETEAKKKETDS-IPQNINALEKDKKLLEVIDAIKKQKQKISENSNKIKEFSGAADT 599
                 ******:: *****  *  *** *.*** ******:*****:*********** *.***:

Sal-1            LKAEVEELKKGIDEDVNKILKPF*------------------------------------ 623
PVP01_0534400    LKAEVEELKKGIDEDVNKILKPF*------------------------------------ 623
Korea            LKAEVEELKKGIDEDQYLKKELQREKYISKNDVAQSYILKIGKLNKGTITYVVEQLRFKK 614
Brazil           LKAEVEELKKGIDEDVNKILELI*------------------------------------ 623
Mauritania       LKAEVEELKKGIDEDVNKILELI*------------------------------------ 622
India            LKAEVEELKKGIDEDVNKILKPF*------------------------------------ 622
Thai             LKAEVEELKKGIDEDVNKILKPF------------------------------------- 622
                 ***************     :
```

```
Sal-1            ------------------------------------------------     623
PVP01_0534400    ------------------------------------------------     623
Korea            MEVPNKVKSIIDAFYNENLQILEDDLAKILCVTSIFITLYSKCYKRN*     661
Brazil           ------------------------------------------------     623
Mauritania       ------------------------------------------------     622
India            ------------------------------------------------     622
Thai             ------------------------------------------------     622
```

Supplemental Table S1. Anti-RBP-2P1 antibody (total IgG), Sex, Age, and parasitemia of 119 infected individuals from Thailand.

Individual's ID	Type of infection*	Antibody (AU)	Sex	Age (yr)	Parasitemia (%)**
VKTS16	clinical	0.0199	F	66	0.208320
VKBT14	clinical	0.2238	M	46	0.014000
VKBT60	clinical	0.0058	M	45	0.015400
VKBT10	clinical	0.0216	M	35	0.017080
VKBT7	clinical	0.1830	M	32	0.022120
VKBT32	clinical	0.0321	F	24	0.028000
VKBT15	clinical	0.0090	M	25	0.038360
VKTS1	clinical	0.0045	M	27	0.045080
VKBT29	clinical	0.1245	F	26	0.054880
VKPR7	clinical	0.0554	F	45	0.064960
VKPR10	clinical	0.0047	M	21	0.076160
VKTS4	clinical	0.0100	M	27	0.083440
VKTS34	clinical	0.2015	M	22	0.096320
VKTS30	clinical	0.1680	M	48	0.127120
VKTS32	clinical	0.0725	F	52	0.163800
VKBT2	clinical	0.2167	F	50	0.057400
VKTS1	clinical	0.0055	M	27	0.045080
VKBT3	clinical	0.0242	M	37	0.073920
VKLS2	clinical	0.0159	M	42	0.075040
VKBT7	clinical	0.2046	M	32	0.022120
VKBT5	clinical	0.0277	F	24	0.201600
VKBT4	clinical	0.0361	M	24	0.080640
VKBT6	clinical	0.0150	M	19	0.054880
VKBT12	clinical	0.0382	M	25	0.217560
VKBT13	clinical	0.0161	M	41	0.061040
VKBT14	clinical	0.2054	M	46	0.014000
VKBT15	clinical	0.0093	M	25	0.038360
VKBT16	clinical	0.0548	F	18	0.088200
VKBT17	clinical	0.1011	M	34	0.156240
VKBT18	clinical	0.1474	M	70	0.011480
VKBT19	clinical	0.0210	M	22	0.008680
VKLS3	clinical	0.0077	M	42	0.017360
VKBT8	clinical	0.0264	M	29	0.028840
VKTS3	clinical	0.0084	M	46	0.287000
VKTS4	clinical	0.0157	M	27	0.083440
VKBT11	clinical	0.0101	M	18	0.005880

VKBT9	clinical	0.0270	F	28	0.293440
VKBT10	clinical	0.0196	M	35	0.017080
VKTS6	clinical	0.0110	M	46	0.304920
VKTS7	clinical	0.2101	M	20	0.139440
VKBT20	clinical	0.0687	F	47	0.039760
VKBT15	clinical	0.0137	M	25	0.038360
VKBT21	clinical	0.0538	M	20	0.054040
VKBT23	clinical	0.0113	M	24	0.067760
VKBT24	clinical	0.0271	F	46	0.015120
VKBT26	clinical	0.0064	M	18	0.164080
VKPR7	clinical	0.0632	F	45	0.064960
VKTS7	clinical	0.0080	M	20	0.139440
VKTS8	clinical	0.0461	F	24	0.054880
VKBT28	clinical	0.0091	M	24	0.041160
VKBT29	clinical	0.1310	F	26	0.054880
VKBT30	clinical	0.0053	M	34	0.017360
VKTS9	clinical	0.0043	F	34	0.025480
VKLS4	clinical	0.0094	M	38	0.176400
VKPR10	clinical	0.0029	M	21	0.076160
VKTS10	clinical	0.0143	M	37	0.283080
VKBT31	clinical	0.0044	F	42	0.138600
VKTS11	clinical	0.0052	M	30	0.155120
VKBT32	clinical	0.0257	F	24	0.028000
VKTS12	clinical	0.0056	F	26	0.022680
VKTS13	clinical	0.0466	M	38	0.068880
VKBT33	clinical	0.1173	F	39	0.140560
VKBT34	clinical	0.0064	F	32	0.207200
VKBT35	clinical	0.0026	M	25	0.377160
VKTS14	clinical	0.0035	F	21	0.033600
VKBT39	clinical	0.0220	M	60	0.156800
VKTS16	clinical	0.0065	F	66	0.208320
VKTS17	clinical	0.0215	M	49	0.181720
VKBT36	clinical	0.0358	F	45	0.069160
VKBT37	clinical	0.0208	F	45	0.070560
VKBT38	clinical	0.1578	M	55	0.073080
VKTS20	clinical	0.0322	F	46	0.264880
VKBT40	clinical	0.0118	F	18	0.040320
VKBT41	clinical	0.0080	M	25	0.167160
VKBT39	clinical	0.0353	M	60	0.156800
VKTS20	clinical	0.0067	F	46	0.264880

VKBT43	clinical	0.0439	M	23	0.019040
VKBT44	clinical	0.0541	M	38	0.014560
VKTS22	clinical	0.0159	F	60	0.015120
VKTS23	clinical	0.1552	M	45	0.017360
VKTS24	clinical	0.0029	M	31	0.018480
VKTS26	clinical	0.0054	M	24	0.123200
VKBT41	clinical	0.0089	M	25	0.167160
VKTS25	clinical	0.0156	M	72	0.182840
VKTS28	clinical	0.0071	M	19	0.442680
VKBT49	clinical	0.0099	M	56	0.096040
VKTS29	clinical	0.2783	F	55	0.053760
VKBT50	clinical	0.1750	M	38	0.055160
VKTS30	clinical	0.1778	M	48	0.127120
VKBT51	clinical	0.0122	M	20	0.019320
VKBT52	clinical	0.0078	M	25	0.330680
VKTS31	clinical	0.0170	F	28	0.164360
VKBT53	clinical	0.0130	M	28	0.171920
VKBT57	clinical	0.0025	F	50	0.145600
VKBT55	clinical	0.0195	M	44	0.039760
VKBT58	clinical	0.0055	M	19	0.231000
VKTS32	clinical	0.0606	F	52	0.163800
VKBT59	clinical	0.0124	M	35	0.144480
VKBT60	clinical	0.0073	M	45	0.015400
N417005	asymptomatic	0.2041	M	18	0.012835
N444001	asymptomatic	0.0063	M	22	0.000626
N372002	asymptomatic	0.0388	M	45	0.000183
N283002	asymptomatic	0.0214	F	29	0.000094
N246109	asymptomatic	0.1747	M	25	0.000133
N272004	asymptomatic	0.0058	M	16	0.000022
N416001	asymptomatic	0.0165	M	56	0.000074
N430002	asymptomatic	0.0185	F	29	0.000077
N440101	asymptomatic	0.0074	M	29	0.000007
N372005	asymptomatic	0.1540	F	32	0.000012
N431001	asymptomatic	0.2146	M	47	0.000046
N242001	asymptomatic	0.1682	M	40	0.000014
N421002	asymptomatic	0.0062	M	56	0.000081
N417002	asymptomatic	0.1464	F	35	0.000023
N380003	asymptomatic	0.1629	M	35	0.000073
N439001	asymptomatic	0.1463	F	40	0.000034
N407001	asymptomatic	0.0165	M	28	0.000009

N405001	asymptomatic	0.0721	M	46	0.000002
N296001	asymptomatic	0.0601	M	18	0.000004
N432002	asymptomatic	0.0829	M	34	0.000132

*Type of infection is classified as *clinical* or *asymptomatic*. Clinical specimens were obtained from symptomatic *P. vivax* malaria patients. Asymptomatic specimens were obtained from asymptomatic *P. vivax* carriers.

**Parasitemia was determined by counting parasites in a 1 µl Giemsa stained thick blood spot for clinical samples, and by qPCR for asymptomatic carriers. For qPCR, parasitemia was estimated based on the genus-specific 18S rRNA gene copy number.
